# Supplementary material for: Reflexive gaze following in common marmoset monkeys
Source: Sci Rep. 2019 Oct 25;9:15292. doi: 10.1038/s41598-019-51783-9 (PMC6814856; doi:10.1038/s41598-019-51783-9)
Supplement: Supplementary file 1 — Supplementary Information [file 41598_2019_51783_MOESM1_ESM.pdf]

Reflexive gaze following in common marmoset monkeys

Silvia Spadacenta[1,*], Peter W. Dicke[1] and Peter Thier[1,*]

[1]Hertie Institute for Clinical Brain Research, Department of Cognitive Neurology, Otfried-Müller-Str. 27, 72076, Tübingen, Germany

*Correspondence: silvia.spadacenta@uni-tuebingen.de

thier@uni-tuebingen.de

SUPPLEMENTARY FIGURES AND TABLES

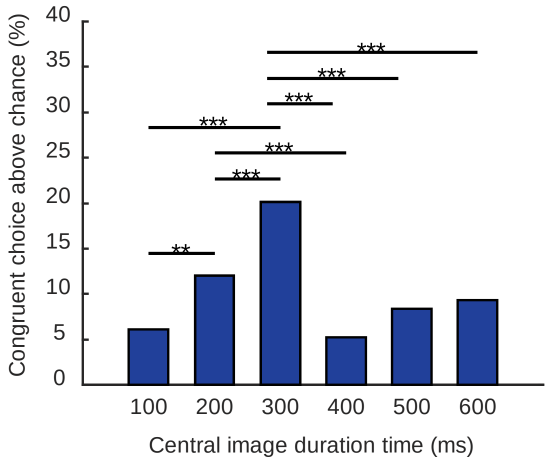

Figure S1. An exposure duration of 300 ms to the oriented face prompts the maximal gaze following

Bar chart of the number of congruent choices above chance level for the data shown in the left panel of figure 1b with statistical comparisons between presentation durations time view based on chi square tests without Yates correction (*** $p < 0.001$; ** $p < 0.01$; only significant comparisons shown). The percentage of congruent choices at 300 ms is significantly larger than for shorter or longer presentation durations.

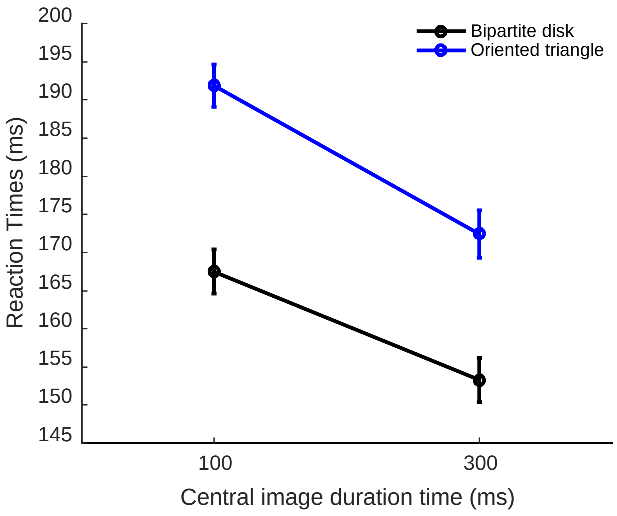

Figure S2. Saccadic reaction times for choices prompted by the bipartite disks and triangles control stimuli

No differences in saccadic reaction times (RT) were registered between choices towards the brighter and darker side (Wilcoxon rank-sum test, 100 ms: $p = 0.8$, zval = 0.235; 300 ms: $p = 0.06$, zval = -1.876). Hence, we pooled the both in order to assess the influence of presentation duration. As for the monochromous disk (see figure 3), RTs decreased with longer exposure to the stimulus (Wilcoxon rank-sum test, $p < 0.001$, zval = 3.776). The same decrease holds for the oriented triangle reaction times ((Wilcoxon rank-sum test, $p < 0.001$, zval = 5.084).

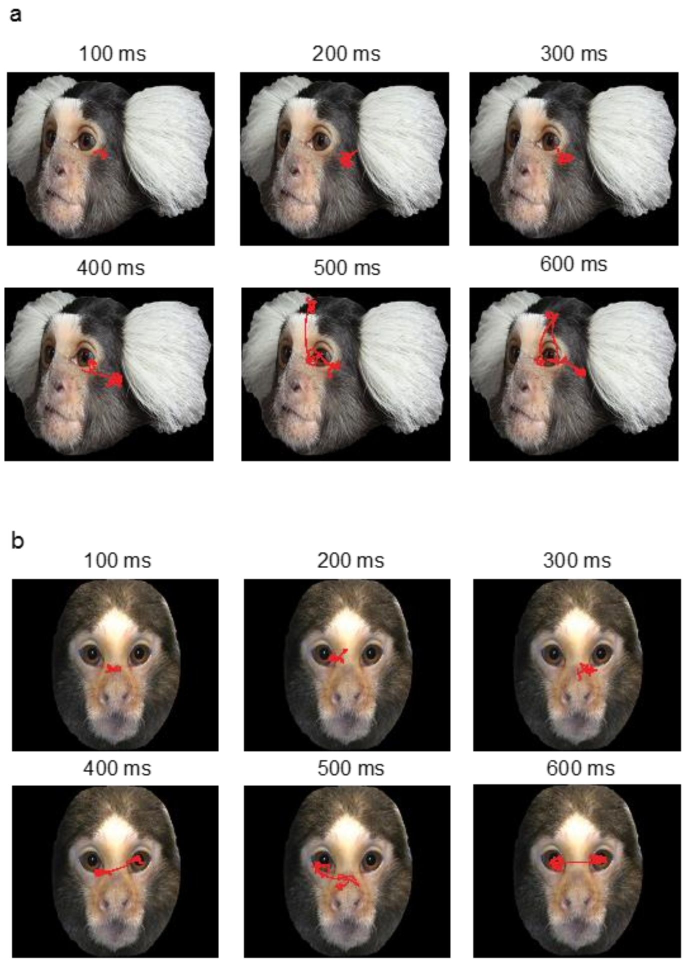

Figure S3. Only longer exposures to the other´s face allow the scrutiny of relevant facial features

Exemplary patterns of eye movement made by the observers when exposed to the oriented face of a conspecific (a) or alternatively to the frontal face of a conspecific lacking the white ear tufts (b) for different durations. Up to 300 ms the eyes of the observer stayed in a small region of the face corresponding to the center of the image, arguably behaviorally not particularly relevant. Only exposure durations of 400 ms and longer allowed exploratory saccades, in these and most other cases aiming at the eye region and only rarely oriented towards the white ear-tufts. The data are from individual sessions with monkey M2.

Table S1. Choice behavior

		GAZE TASK		CONTROL TASKS											
		Face left	Face right	Direct gaze		Disk		Bipartite disk (black half left)		Bipartite disk (black half right)		Triangle left		Triangle right	
	Image duration (ms)	% correct		% left choice	% right choice	% left choice	% right choice	% left choice	% right choice	% left choice	% right choice	% left choice	% right choice	% left choice	% right choice
M1	100	76.98	38.46	64.18	35.82	69.11	30.89	44.17	55.83	55.56	44.44	52.63	47.37	50.00	50.00
	200	75.37	53.73	55.66	44.34	57.51	42.49								
	300	81.38	71.03	57.58	42.42	53.25	46.75	56.46	43.54	41.45	58.55	49.17	50.83	45.45	54.55
	400	56.69	57.25	51.71	48.29	57.47	42.53								
	500	56.64	43.42	54.41	45.59	60.75	39.25								
	600	50.77	53.73	44.28	55.72	50.75	49.25								
M2	100	60.71	53.96	66.38	33.62	67.54	32.46	53.13	46.88	61.84	38.16	60.74	39.26	52.76	47.24
	200	70.86	52.98	57.00	43.00	63.39	36.61								
	300	72.39	60.14	55.27	44.73	56.62	43.38	62.50	37.50	43.66	56.34	47.40	52.60	46.50	53.50
	400	62.67	43.54	59.81	40.19	55.14	44.86								
	500	68.85	51.49	56.60	43.40	58.64	41.36								
	600	70.97	46.71	65.83	34.17	56.08	43.92								
M3	100	45.33	63.82	43.24	56.76	45.33	54.67	41.49	58.51	70.33	29.67	49.66	50.34	42.86	57.14
	200	49.63	70.40	35.71	64.29	39.83	60.17								
	300	56.95	78.62	43.44	56.56	50.24	49.76	34.78	65.22	59.26	40.74	41.77	58.23	45.52	54.48
	400	50.43	61.29	44.90	55.10	46.12	53.88								
	500	53.68	76.82	37.74	62.26	40.59	59.41								
	600	53.70	79.23	33.50	66.50	38.25	61.75								

Table S2. Reaction times: gaze and control tasks (direct gaze and monochromous disk stimuli)

			GAZE TASK				CONTROL TASKS				
			Left Face		Right Face			Direct gaze		Disk	
	Image duration (ms)		Mean	Se	Mean	Se		Mean	Se	Mean	Se
**M1**	100	Correct	123.36	6.61	135.30	9.64	Left	164.53	6.39	156.11	4.19
		Wrong	143.00	13.85	139.88	6.47	Right	165.81	7.39	163.78	7.21
	200	Correct	136.51	6.72	134.56	7.70	Left	149.64	5.13	154.96	5.33
		Wrong	152.55	8.65	140.63	9.12	Right	130.76	4.37	142.15	8.38
	300	Correct	128.31	6.42	110.62	4.83	Left	150.77	5.88	140.94	4.97
		Wrong	135.78	14.57	121.95	9.40	Right	141.87	5.79	140.96	6.37
	400	Correct	142.88	8.37	128.76	7.63	Left	132.53	5.19	132.94	4.47
		Wrong	131.65	8.65	158.04	10.67	Right	124.79	4.24	130.12	6.74
	500	Correct	135.02	8.46	133.11	6.24	Left	135.85	5.53	145.41	5.25
		Wrong	132.95	5.91	137.18	6.17	Right	138.67	5.37	128.27	5.82
	600	Correct	127.95	6.81	127.86	6.56	Left	148.67	5.24	140.01	6.28
		Wrong	132.98	6.53	146.16	7.57	Right	137.84	4.61	135.07	6.59
**M2**	100	Correct	145.62	11.25	111.31	10.34	Left	160.91	6.22	151.10	4.76
		Wrong	160.56	10.44	170.31	9.43	Right	165.04	9.41	148.85	8.12
	200	Correct	143.15	8.11	122.51	8.98	Left	158.85	7.93	163.72	6.05
		Wrong	174.77	15.91	184.32	9.96	Right	117.87	8.15	116.85	7.56
	300	Correct	166.27	9.19	157.64	9.33	Left	169.29	7.27	136.16	6.59
		Wrong	147.38	13.78	181.75	11.76	Right	151.29	7.94	118.76	7.77
	400	Correct	153.90	9.97	155.59	9.36	Left	178.25	7.56	167.63	7.09
		Wrong	194.00	10.35	168.90	9.54	Right	146.74	8.24	127.73	8.50
	500	Correct	182.46	10.44	140.68	10.56	Left	185.46	6.51	145.19	8.31
		Wrong	176.21	12.10	174.14	9.48	Right	183.46	7.72	123.42	7.50
	600	Correct	169.75	8.22	145.93	7.95	Left	177.28	6.65	156.70	8.75
		Wrong	182.58	11.67	170.86	7.96	Right	169.25	8.75	133.27	7.39
**M3**	100	Correct	161.90	12.90	162.26	8.96	Left	203.49	9.88	184.96	9.30
		Wrong	198.57	9.30	214.43	12.74	Right	194.33	8.09	206.23	8.52
	200	Correct	212.19	14.38	207.75	10.19	Left	227.61	10.99	186.05	11.52
		Wrong	203.81	10.17	221.05	16.26	Right	212.24	8.43	178.01	7.14
	300	Correct	211.23	12.99	223.13	8.05	Left	223.07	9.49	192.73	10.99
		Wrong	219.08	11.97	216.62	15.85	Right	228.70	7.14	190.96	7.89
	400	Correct	220.29	12.39	193.92	8.45	Left	206.20	11.38	173.30	10.53
		Wrong	222.14	11.09	194.45	14.57	Right	204.46	8.41	191.45	9.14
	500	Correct	206.97	12.55	177.48	7.71	Left	226.75	10.44	181.06	11.95
		Wrong	205.48	9.76	184.88	19.20	Right	217.09	6.75	199.06	9.33
	600	Correct	193.19	14.04	198.71	7.46	Left	216.32	13.06	171.51	13.74
		Wrong	206.16	10.58	210.22	21.80	Right	212.96	6.59	194.58	8.48

Table S3. Reaction times: control task (bipartite disk and triangle stimuli)

			CONTROL TASKS							
			Bipartite disk (black half left)		Bipartite disk (black half right)		Triangle left		Triangle right	
	Image duration (ms)		Mean	Se	Mean	Se	Mean	Se	Mean	Se
**M1**	100	Correct	163.99	7.63	154.67	8.52	184.38	12.54	161.89	10.73
		Wrong	140.36	8.23	165.63	10.67	154.69	11.26	176.47	11.41
	300	Correct	131.81	7.23	158.70	10.21	145.69	10.81	131.04	11.10
		Wrong	134.46	7.18	130.13	8.20	137.4	10.34	136.94	8.85
**M2**	100	Correct	127.47	7.81	173.79	6.15	179.36	7.25	188.93	9.22
		Wrong	158.74	8.57	150.90	9.65	212.15	10.08	195.92	8.41
	300	Correct	128.48	10.37	158.74	8.57	190.80	9.27	169.96	10.64
		Wrong	149.78	7.35	127.47	7.81	143.09	10.03	178.29	12.81
**M3**	100	Correct	205.48	13.18	186.67	9.31	203.99	9.48	208.94	9.78
		Wrong	130.44	9.85	213.19	18.48	181.69	9.11	188.57	10.96
	300	Correct	186.95	11.89	187.58	11.92	191.70	12.40	209.38	10.57
		Wrong	138.09	12.09	221.79	17.46	186.79	8.92	215.91	12.36
